# Protease-resistant SOD1 aggregates in amyotrophic lateral sclerosis demonstrated by paraffin-embedded tissue (PET) blot

**DOI:** 10.1186/s40478-014-0130-x

**Published:** 2014-08-28

**Authors:** Petra Steinacker, Christian Berner, Dietmar R Thal, Johannes Attems, Albert C Ludolph, Markus Otto

**Affiliations:** Department of Neurology, University of Ulm, Oberer Eselsberg 45, 89081 Ulm, Germany; Institute of Pathology - Laboratory of Neuropathology, University of Ulm, Ulm, Germany; Institute for Ageing and Health, Newcastle University, Newcastle upon Tyne, UK

**Keywords:** Amyotrophic lateral sclerosis, Superoxide dismutase 1, Protein aggregates, p62, Ubiquitin

## Abstract

**Objectives:**

The paraffin-embedded tissue (PET) blot technique followed by limited protease digestion has been established to detect protein aggregates in prion diseases, alpha-synucleopathies, and tauopathies. We analyzed whether the scope of the method can be extended to analyze aggregates in mouse and human tissue with amyotrophic lateral sclerosis (ALS) associated with superoxide dismutase 1 (SOD1) mutation.

**Methods:**

Formalin-fixed and paraffin-embedded brain and spinal cord tissue from SOD1^G93A^ mice was first analyzed for the expression of SOD1, aggregated SOD1, ubiquitin, and p62 by convential immunohistochemistry and then used to establish the PET blot technique, limited protease digest, and immunodetection of SOD1 aggregates. The method was then transferred to spinal cord from an ALS patient with SOD1^E100G^ mutation.

**Results:**

Mouse and human paraffin-embedded brain and spinal cord tissue can be blotted to membranes and stained with anti-SOD1 antibodies. The SOD1 labelling is abolished after limited proteolytic digest in controls, whereas under identical conditions SOD1 aggregates are detected the SOD1^G93A^ mouse model of ALS and in human familial ALS. The most prominent areas where aggregates could be detected are the brainstem and the anterior horn of the spinal cord.

**Discussion:**

Applicability of the PET blot technique to demonstrate SOD1 aggregates in ALS tissue associated with mutations in the SOD1 gene offers a new approach to examine potential spreading of aggregates in the course of ALS.

## Introduction

Neurodegenerative diseases are characterized by the accumulation of proteins with aberrant conformation in the affected tissues. Disturbances of mechanisms that normally ensure clearance of misfolded proteins have been identified to underlie or contribute the deposition. Included here are e.g. dysfunction of the chaperone machinery, failure of the proteasomal system [1,2], and impairment of autophagic mechanisms [3]. Recently, it was recognized that template assisted misfolding or seeded polymerization contributes to the burden of protein aggregates in a number of proteinopathies, comparable to the propagation of conformationally changed prion protein in prion diseases [4,5].

Amyotrophic lateral sclerosis (ALS) is a phenotypically heterogeneous disease characterized by degeneration of upper and lower motoneurons. 90% of ALS cases are sporadic and 10% are caused by mutations in different genes. Around 20% of the familial cases are associated with mutations in the Cu/Zn superoxide dismutase 1 (SOD1) [6]. Mutated SOD1 forms aggregates of fibrillar structure in the affected tissues [7,8] and recent data suggest that there is also a propensity for aggregation of posttranslationally modified wild type SOD1 in a subset of sporadic ALS cases [9]. Misfolding and aggregation of SOD1 in the spinal cord and the motor cortex is also observed in mice transgenic for disease associated mutated human SOD1 representing the most established model for ALS [10]. Neuronal inclusions are positive for ubiquitin and p62 [11,12].

The misfolding state depends on the mutation and on the method of detection [13]. Some antibodies were generated and are in part commercially available which are reported to solely recognize misfolded SOD1. A way to detect misfolded proteins independently on antibodies recognizing the misfolded form is to subject tissue or homogenates from animals or humans with a proteinopathy to a limited protease digest, leading to destruction of the physiologically folded protein but leaving off the conformationally changed form(s), which afterwards can be immunodetected by non-conformation-specific antibodies. A method that was initially established for the detection of misfolded prion protein in fixed tissue from patients or animals with prion disease [14,15] is the paraffin-embedded tissue (PET) blot technique. This technique combines the advantages from highly sensitive immunohistochemistry, reveals anatomical resolution and can be performed with paraffin-embedded tissue.

Here we report on the applicability of the PET blot technique for the detection of misfolded SOD1 in paraffin-embedded tissue from ALS mice and from human fALS post-mortem tissue.

## Material and methods

### Mice breeding

The principles of laboratory animal care were followed and use of the mice was performed in accordance with the national guidelines and was approved by local authorities. Male mice transgenic for human SOD1^G93A^ (huSOD1G93A, B6.Cg-Tg[SOD1-G93A]1Gur/J; stock number 004435) were acquired from the Jackson laboratory (Ben Harbor, Maine). These transgenic mice are characterized by survival times of 128.9+/−9.1 days. By breeding with female C57Bl/6J from an in-house breed wild type mice and SOD1^G93A^ littermates of either sex were generated for the experiments. At terminal disease state the mice used for establishment of the PET-blot were between 132 and 159 days old.

### Tissue preparation

Mice at terminal disease stage were anesthetized and transcardially perfused with PBS for cryoconservation of the tissue or by PBS followed by paraformaldehyde in case of subsequent paraffin embedding as published elsewhere [16]. Prepared were the spinal cords as well as the whole brains separated sagittally into the two hemispheres.

The study of human control tissue was performed in compliance with university ethics committee guidelines and the laws governing human tissue usage in the German federal state of Baden-Württemberg. Consent for autopsy was obtained for all patients and controls or from their next of kin. Brain tissue of the ALS case was collected by the Newcastle Brain Tissue Resource (NBTR) at Newcastle University, UK, after relevant informed consent from the donor and in accordance with Newcastle University ethics board and ethical approval awarded by The Joint Ethics Committee of Newcastle and North Tyneside Health Authority (reference: 08/H0906/136).

Two control cases were studied: 1. male, 38 years of age, head trauma, (neuropathological staging: Braak NFT 0 [17], Abeta phase 0 [18], Braak PD stage 0 [19]); 2. female 72 years of age, ruptured aortic aneurysm (neuropathological staging: Braak NFT I, Abeta phase 0, Braak PD stage 0.

The ALS case was a female age 48 at death who had the diagnosis 12 years before death. The mother, two uncles and a sister were also affected by ALS. Testing during lifetime revealed the E100G mutation in the SOD1 gene. Initial symptoms were cramp and muscle twitching in legs, progressing to fasciculation in upper limbs and trunk and lower limb weakness. Marked bulbar symptoms developed. There was no apparent cognitive impairment. The cause of death was cardiac arrest and asphyxia secondary to ALS (neuropathological staging: Braak stage 0, CERAD score [20] none, no Lewy Body pathology, no cerebral infarcts).

The spinal cords were removed in toto and fixed in a 4% aqueous solution of formaldehyde for 10–20 days prior to dissection. The dura mater of each spinal cord was then opened with a dissecting scissors and anchored bilaterally with pins to a cork board. After macroscopic inspection, tissue blocks from the cervical, thoracic, lumbar, and sacral spinal cord with the dorsal and ventral roots were made, embedded in paraffin, and sectioned at 7 μm in the transversal plane.

### Immunohistochemistry

Paraffin-embedded tissue sections (5 μm) were dried over night at 37°C and immersed in two changes of 5 min xylene and acetone. Deparaffinized sections were rinsed with water, exposed to microwave and afterwards endogenous peroxidase was blocked with 3% hydrogen peroxide in methanol. After 2 washes with PBS for 5 min the sections were perfused with 0.2% Triton in PBS and then blocked with 10% fetal bovine serum PBS. Subsequently, the respective primary antibody was diluted in blocking solution (polyclonal anti-human SOD1 (abcam ab52950) and anti human/mouse SOD1 antibodies (ab16831), 1:500; anti-ubiquitin, Biorad 9400–1004, 1:250, monoclonal anti-p62 (SQSTM1), abcam ab91526, 1:500) and applied over night at 4°C. After 3 washes in PBS for 5 min the sections were either incubated for 1 h at RT with goat-anti-rabbit peroxidase-coupled secondary antibody (Biorad), washed again 3 times and developed with DAB (sigma fast) for about 10 min, or incubated with biotin-coupled secondary anti-mouse antibody (Biorad), washed again and developed with ABC-kit (Pierce). Counterstaining with 100% hemalaun was followed by dehydration and mounting of the slices with entellan.

### Paraffin-embedded tissue blot

Pieces of PVDF membrane (20 mm x 50 mm Fluorobind, Serva, Heidelberg, Germany) were prewetted in methanol for 10 sec followed by water. Sagittal mouse brain sections of 5 μm or transverse mouse/human spinal cord sections were cut and put into a water bath at 40°C, transferred to the prewetted membrane and dehydrated at 55°C for 2 h. Deparaffination/rehydration was carried out by two changes of 5 in: Xylene, 100% - 96% - 70% ethanol, TBS (50 mM Tris–HCl pH 7.6, 150 mM NaCl) including drying of the membrane between xylene and 100% ethanol and between 70% ethanol and TBS which avoids detaching of the membrane from the glas.

For the establishment of partial proteinase K (PK) digest a series of experiments was carried out applying 5-250 μg/ml PK for 4-21 h. 5 μg/ml PK and a digestion time of 15 h was worked out to be best: PK below 5 μg/ml or digestion times below 15 h yielded results comparable to PK-untreated PET-blots and increases in concentration or digestion times above 5 μg/ml 15 h yielded blank PET-blots. All PK digested PET blots shown were treated with 5 μg/ml PK added to digest buffer (10 mM Tris–HCl pH 7.8, 100 mM NaCl, 0.1% Brij) and incubated with the membrane in an oven at 55°C for 15 hours. After the digest the PET-Blots were washed 3 × 5 min in TBS with 0.5% Tween 20 (TBS-T).

The endogenous peroxidase was blocked at RT by incubation with 3% hydrogen peroxide for 5 min. Then the sections were washed three times with TBS-T for 5 min. Epitope retrieval was achieved by incubation of the membrane with 4 M guanidiumthiocyanat in 10 mM Tris–HCl, ph 7.8, for 15 min. After 3 washes (TBS-T 5 min) the blots were blocked (Tropix I-Block, Applied biosystems, Bedford, U.S.A) for 1 h at RT. Primary antibodies specific for human SOD1 or for mouse&human SOD1 (both from Abcam,see above)), for 14-3-3 proteins (Santa Cruz sc-629), were diluted 1:1000 in I-Block, respectively, and incubated with the blots on a rocker over night at 4°C. Biotinylated secondary antibody (Dako, Glostrup, Denmark) diluted 1:500 in I-Block was added after 3x 10 min washes with TBS-T for 1 h at RT. Excess antibody was washed out 5 times with TBS-T for 10 min. Subsequently the membranes were incubated avidin-biotinylated-complex (ABC) solution for 1.5 hours. After 5 x 10 min wash in TBS-T the blots were developed by adding 3,3' diaminobenzidine (fast DAB, Sigma, Steinheim, Germany) for 10 min. The membranes were mounted on glass slides with a 3:2 ratio of DMSO:96% ethanol.

Pictures were taken with a Zeiss Mirax digital scanning microscope, a Zeiss Axiovert 200 or Axioskop 2plus microscope, post processing was carried out using adobe photoshop.

## Results

**Figure 1:**
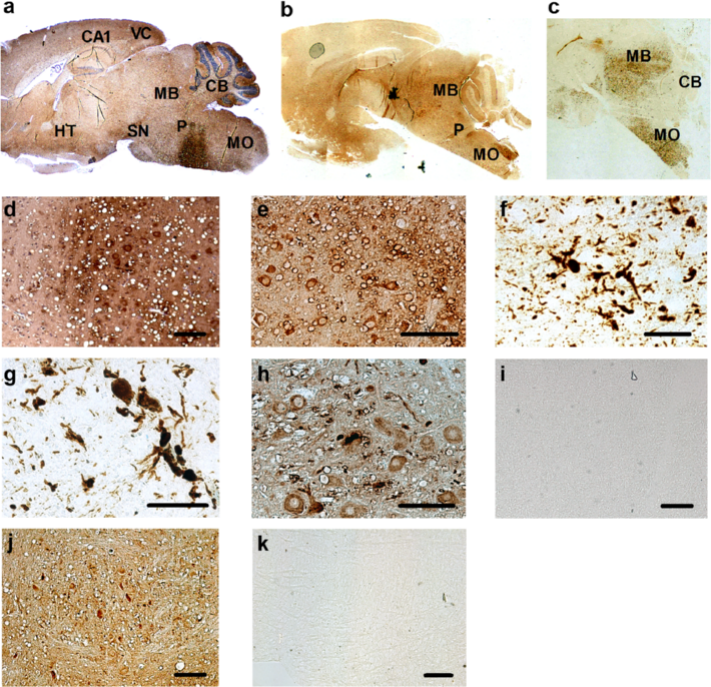
**Sagittal brain slices from a SOD1** ^**G93A**^ **transgenic mouse at terminal disease stage and from control wild type mice. (a)** Immunohistochemistry applying anti-human SOD1 antibody (counterstaining: HE). Most prominent expression can be observed in motor-associated regions of the pons and the medulla as well as in midbrain regions, e.g. substantia nigra pars compacta and the reticular nucleus. A pronounced staining of the cerebellar molecular layer and a moderate staining of cerebellar nuclei can be seen. The Ammon’s horn CA1 region as well as the subiculum is temperately stained. In a detailed view of the medulla oblongata after conventional IHC of human SOD1 in the transgenic mouse brain tissue intense neuronal cytoplasmic staining as well as staining of the neuropil is visible **(d)**. The edges of pathologic vacuoles are also frequently stained. In **(b)** a PET-blot of a sagittal brain slice from a SOD1^G93A^ mouse after staining of human SOD1 is shown. A relatively homogeneous overall staining can be seen with areas of intense staining mainly in the brain stem. The staining is weaker compared to conventional IHC however the cytoplasmic as well as vacuole staining is preserved as it is shown at higher magnification of the medulla oblongata **(e)**. When subjecting a PET-blot of a SOD1^G93A^ mouse to limited protease digestion the residual tissue can be recognized and staining is left in the brain stem **(c)**, appearing as large SOD1-positive clumps that often have skein-like structure, which is shown for the midbrain **(f)** and medulla oblongata **(g)**. Controls are shown in **(h-k)**: PET-blot of a sagittal WT mouse brain section and immunodetection with an antibody against mouse SOD1 without PK treatment **(h)** and after PK **(i)** shows that the endogenous SOD1 is not resistant to PK digestion. PET-blot of a WT mouse brain slice and immunodetection of 14-3-3 proteins without **(j)** and after PK digest **(k)** shows that even the highly expressed 14-3-3 is quantitatively degraded. Bars represent 200 μm **(k)**, 100 μm **(d, e, h, i, j)**, and 50 μm **(f, g)**.

In brain sections from end-stage SOD1^G93A^ mice immunohistochemically (IHC) stained for human SOD1 a widespread expression of the transgene is visible with most intense staining in the brain stem, particularly in motor-associated regions of the pons and the medulla as well as in midbrain regions, e.g. substantia nigra pars compacta and the reticular nucleus (Figure 1a). A pronounced staining of the cerebellar molecular layer and a moderate staining of cerebellar nuclei can be seen. At higher magnification, the strong staining of the cytoplasm of neurons in the medulla oblongata (Figure 1d) and in the midbrain area becomes visible.

In PET-blots applying anti-human SOD1 antibody (Figure 1b) a less intense overall staining is observed which appears to be more uniformly distributed over the whole tissue compared to the IHC. Taking a closer look distinct staining becomes visible frequently localized to the edges of pathologic vacuoles as well as to the cytoplasm of neurons in both the medulla oblongata (Figure 1e) and the midbrain (not shown). When PET blots are digested with proteinase K at a concentration of 5 μg/ml for 15 h the edges of the blotted tissues are barely seen and immunopositive structures are left almost entirely in the brain stem (Figure 1c). The structures that are strongly labelled have a size of few to around 30 μm and round or (tangled) skein-like shape (Figure 1f,g). Cellular structures are mostly deleted.

In contrast, staining for endogenous murine SOD1 in PET-blots of wild type (WT) mice brain sections which appears relatively uniformly distributed over the blot with predominantly cytoplasmic staining as shown for the medulla region (Figure 1h) does not resist proteolytic digestion with 5 μg/ml protease K for 15 h (Figure 1i). PET-blots from WT mice brains were also probed with anti-human SOD1 anibodies yielding no staining (data not shown) verifying the specificity of antibody binding in analyses of transgenic tissue.

Brain areas that are strongly positive for transgenic SOD1 are frequently also positive in PK digested PET-blots. To exclude that the SOD1 labelling that can be detected after limited PK digestion is only leftover because of initially high expression levels, we did experiments applying the PET-blot technique to another highly expressed protein in the brain, the 14-3-3 protein. Without proteolysis an intense cytoplasmic staining can be seen (Figure 1j). By treatment of the PET-blot with PK staining is completely abolished in the medulla as well as in other brain regions and only faint transparent tissue derived structures are visible (Figure 1k).

**Figure 2:**
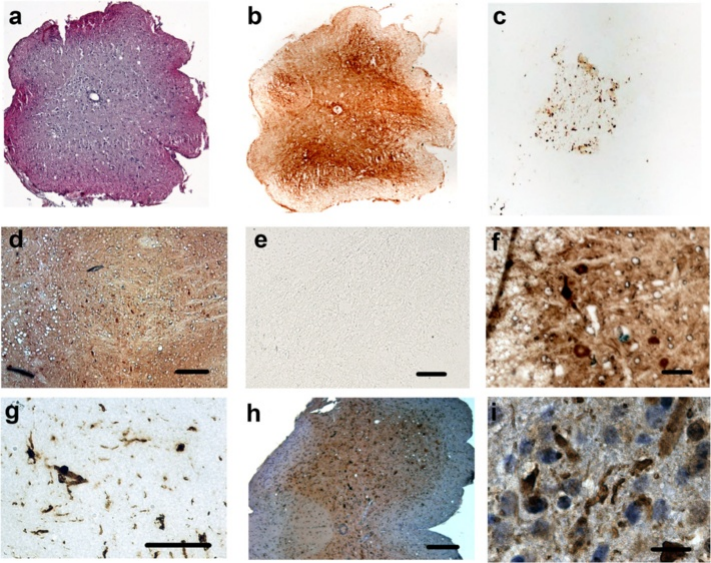
**Transversal spinal cord sections (7 μm) from SOD1** ^**G93A**^ **transgenic mice at terminal disease state and from control mice. (a)** HE-stained section of a SOD1^G93A^ mouse spinal cord. **(b)** Immunostaining of human SOD1 with PET-blot technique without proteolysis reveals strong staining of the grey matter of a SOD1G93A spinal cord. If the PET-blot is treated with PK labelling of grey matter area is left **(c)**. In the PET-blot of control tissue from a wild type mouse also strong labelling applying an antibody recognizing endogenous SOD1 observed **(d)** which is completely abolished by limited proteolysis **(e)**. Higher magnification of the PET blots in b and c revealed the pronounced cellular staining in the ventral horn area of the untreated spinal cord PET-blot **(f)**. In the PK treated PET-blot large numbers of aggregates of different size and shape are detected **(g)**. Staining with an antibody against ubiquitin is shown in an overview **(h)** and at higher magnification **(i)** for comparison. Bars represent 20 μm **(i)**, 50 μm **(g)**, 100 μm **(f)**, and 200 μm **(d, e, h)**.

The PET-blot technique can also be applied to transversal mouse spinal cord sections (HE-stained section in Figure 2a). High expression of the transgenic human SOD1 is detected by anti-human SOD1 antibodies in the cells in the grey matter (Figure 2b) and an intense staining of structures in the grey matter is even obtained after limited proteolytic digest (Figure 2c). This is in contrast to mouse WT tissue: the staining of PET- blots obtained after probing anti-mouse SOD1 antibodies (Figure 2d) is abolished by PK treatment (Figure 2e). The labelled structures in the SOD1^G93A^ spinal cord appear skein-like or tangled (Figure 2g) as in the brain stem and can hardly be seen in conventional SOD1 immunohistochemistry (data not shown) or in PK-untreated SOD1 PET-blots (Figure 2f). Also in ubiquitin IHC (Figure 2h,i) large labelled structures are observed but less frequently and they are more difficult to recognize than in the protease treated SOD1 PET-blot.

**Figure 3:**
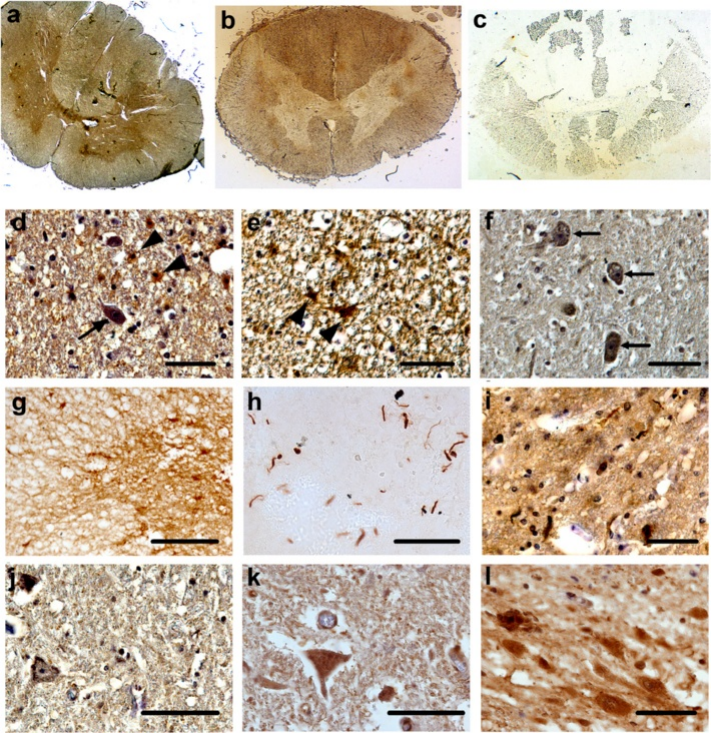
**Transverse spinal cord sections from an ALS Patient with SOD1** ^**E100G**^ **mutation and sections from a control patient. (a)** shows a SOD1 IHC, **(b)** a SOD1 PET-blot and **(c)** a protease treated SOD1-PET blot. In the IHC shown in more detail in d-e staining of neurons and glial cells is visible in the gray matter (arrows and arrow heads in d, respectively). **(e)** shows that glial cells in the white matter are also stained. **(f)** In the control spinal cord section SOD1 is only detected in neurons of the gray matter. In PET-blots SOD1 is labelled in the grey matter as well as in glial cells in the white matter **(g)**. However, in PK treated PET-blots aggregates are strongly labelled in the grey matter area **(h)**. Aggregates can only occasionally be seen in p62 IHC (i: ALS, j: control) and are undetectable in ubiquitin IHC (k: control, l: ALS). Bars represent 50 μm **(a-f, h-l)** and 100 μm **(g)**.

We went then to the examination if also human post-mortem ALS tissue is suitable for the PET-blot analysis of SOD1 aggregates. In transverse spinal cord sections from an ALS patient with the SOD1^E100G^ mutation we found by SOD1 IHC (overview in Figure 3a) expression in both neurons and glial cells in the grey matter (Figure 3d) and glial cells in the white matter (Figure 3e). In the spinal cord section of a control patient SOD1 is found in neurons of the grey matter (Figure 3f) but glial cells are largely negative. Aggregates in ALS tissue cannot be obviously seen, when SOD1 is stained. We performed IHC for ubiquitin and for p62. While the ubiquitin IHC was indistinguishable comparing ALS with control tissue (Figure 3k,l), neuropathological differences can be seen in the ALS tissue stained for p62, namely occasionally occurring aggregate-like structures (Figure 3i,j).

When human ALS spinal cord tissue is subjected to PET-blot SOD1 staining in the white and grey matter is preserved (Figure 3b,g), however aggregates become clearly visible when the blotted tissue is PK-treated before immunodetection (Figure 3c,h).

## Discussion

In recent years evidence accumulated that in neurodegenerative diseases, including ALS, disease progression is caused or paralleled by spreading of the disease specific respective pathological protein. Comparable to prion diseases, in which the pathological prion protein propagates by a seeding/nucleation polymerization process and is transferred from cell to cell, stereotypic spread has been observed in e.g. synucleopathies and Alzheimer’s disease [21–24]. For *in vitro* and *in vivo* studies and especially for the examination of the neuropathologically characteristic misfolded, oligomerized and aggregated proteins discrimination between normal and aberrant forms is essential.

The PET-blot approach differentiates between normal and aggregated proteins. It takes advantage of a limited protease resistance of conformationally changed proteins compared to the normal forms in tissue sections blotted onto PVDF or nitrocellulose membranes prior to immunodetection. This was first established for prion diseases and the PK resistance was attributed to the amyloid structure with high beta sheet content of the pathological prion protein [15]. The PET-blot technique, which combines high sensitivity of detection with acceptable histological resolution, has been shown to be applicable to study aggregate distribution and follow the spreading also in synucleopathies and Alzheimer’s disease [25,26]. This data tempted us to test if SOD1 aggregates can also be detected in PET-blots of brain sections from genetic ALS brains. Especially for the study of post-mortem paraffin-embedded human ALS brains and spinal cords collected in biobanks as well as for the longitudinal study of ALS mouse tissue the development of approaches to track aggregates during disease progression is important.

Here we show that brain and spinal cord tissue from SOD1^G93A^ mice and from a patient with SOD1 mutation can be subjected to the PET-blot method and that aggregates resist a limited protease digest while endogenous/normal forms of SOD1 are degraded and not longer detected by conventional SOD1 antibody. Compared to IHC staining of ubiquitin and p62, both of which are published to bind SOD1 aggregates and are used to study diseased tissue [27–29], aggregates can easier be seen and a reliable quantification seems possible.

Recently, antibodies specifically recognizing misfolded SOD1 have been developed and successfully used for examination of human and mouse ALS tissue. Some of these antibodies also react with conformationally changed post-translationally modified wild-type SOD1 forms present also in the majority of sALS cases revealing inclusions that weren’t seen formally presumably because of masking by the abundant properly folded SOD1 [9,30–32]. It has to be demonstrated if it is possible to differentiate between different misfolded SOD1 forms with the PET-blot approach, e.g. by applying a range of proteinase concentrations, however masking of aggregates by reactivity of the antibody to alternative SOD1 forms is not to be expected. Future experiments have to be conducted to analyse SOD1 aggregates by IHC with conformation dependent antibodies in order to compare the results with those that came up from PET-blots. Beyond that the analysis of PET-blots with conformation dependent antibodies will probably extent the detection potential and knowledge about the behaviour of muSOD1 aggregates during progression of disease and even of misfolded WT SOD1 in sporadic ALS.

Different mutations in the SOD1 gene can be associated with different properties of the protein with regard to hydrophobicity, di- and oligomerization, aggregation propensity, and toxicity [33–35]. Localization and tracking of mutated SOD1 offers the opportunity to examine several aspects of ALS, as selective vulnerability of specific cells, cell to cell transmission, or co-occurrence of inclusions and neurodegeneration. Exemplarily for human fALS, we analyzed fixed spinal cord tissue from a patient with the E100G mutation in the SOD1 gene. For this mutation posterior column involvement has been reported and ubiquitinated neuronal inclusions have been infrequently detected [36]. In the spinal cord subjected to PET-blot aggregates are easily investigatable and can be localized to areas of the grey matter.

In conclusion, we established a technique for the analysis of misfolded SOD1 in familial ALS useful in aggregate detection, localization and tracking that is applicable to biobanked material. Subgroups of ALS might be identified with different propagation of conformationally changed SOD1.
